# Feeling good in old age: factors explaining health-related quality of life

**DOI:** 10.1186/s12955-018-0877-z

**Published:** 2018-03-13

**Authors:** Manuela Alcañiz, Aïda Solé-Auró

**Affiliations:** 1 Riskcenter, Department of Econometrics, Statistics and Applied Economy, Universitat de Barcelona, Av. Diagonal 690, 08034 Barcelona, Spain; 2 Faculty of Economics and Business, Universitat Oberta de Catalunya, Av. Tibidabo 39-43, 08035 Barcelona, Spain; 3 DemoSoc Research Group, Department of Political and Social Sciences, Universitat Pompeu Fabra, C/ Ramon Trias Fargas, 25-27, 08005 Barcelona, Spain

**Keywords:** Oldest-old population, Health-related quality of life, EuroQol-5D, Spain

## Abstract

**Background:**

Sustained growth in longevity raises questions as to why some individuals report a good quality of life in older ages, while others seem to suffer more markedly the effects of natural deterioration. Health-related quality of life (HRQL) is mediated by several easily measurable factors, including socio-demographics, morbidity, functional status and lifestyles. This study seeks to further our knowledge of these factors in order to outline a profile of the population at greater risk of poor ageing, and to identify those attributes that might be modified during younger stages of the life course.

**Methods:**

We use nationally representative data for Catalonia (Spain) to explain the HRQL of the population aged 80-plus. Cross-sectional data from 2011 to 2016 were provided by an official face-to-face survey. HRQL was measured using EQ-VAS – the EuroQol-5D visual analogue scale – which summarizes current self-perceived health. Multivariate linear regression was used to identify variables influencing the EQ-VAS score.

**Results:**

Sociodemographic factors, including being older, female, poorly educated and belonging to a low social class, were related with poor HRQL at advanced ages. The presence of severe mobility problems, pain/discomfort, and anxiety/depression were highly correlated to the HRQL of the elderly, while problems of self-care and with usual activities had a weaker association.

**Conclusions:**

Encouraging the young to stay in education, as well as to adopt healthier lifestyles across the lifespan, might ensure better HRQL when individuals reach old age. More multidisciplinary research is required to understand the multifaceted nature of quality of life in the oldest-old population.

## Background

Life expectancy today is higher than ever before, with the modal length of life in low-mortality regions approaching 91 years for women and 86 years for men [1]. At the global scale, low- and middle-income countries are recording large declines in mortality at younger ages [2], while in high-income countries the gains in life expectancy are due mainly to the decreasing trends in mortality rates among the elderly [3]. Although in these more advanced economies current life expectancy at older ages reflects current mortality conditions and reaching the fourth age has become a relatively normal ageing phenomenon, exposure to different conditions across the lifespan results in lower rates of survival to old age for earlier- than for later-born cohorts [4]. Nevertheless, the ‘long-lived’ population continues to grow rapidly in many countries thanks to increasing rates of survival at all ages, particularly among the elderly, and past fertility trends [5].

Adding years to the lifespan gives rise to new challenges, as the dynamics of health in older age are both complex and require addressing from a multidisciplinary perspective [6, 7]. While living a long life in good health is a universal aim, ageing is a multifaceted, long-term process that is not faced with equal success by all individuals [8]. A consideration of only the specific diseases or limitations that might affect an older person would be an overly simplistic approach to take here, given that what is required is an evaluation of their impact on trajectories of health and functioning [9]. Ultimately, it is assessments of the quality of life (QoL) that are indicative of poor or successful ageing processes [10].

QoL is neither readily defined nor operationalized given its multidimensional nature [11]. One of its most critical dimensions can be identified as health-related quality of life (HRQL), which is concerned with the effects of health, illness and treatment on QoL, but which excludes cultural, political or societal attributes of life. There is an extensive literature describing different methods for measuring HRQL [12–14], but some authors [15] identify two main types. The first is that of direct elicitation, which includes such methods as time trade-off and rating scales. The time trade-off method involves asking subjects to imagine having to live in a given state of poor health for “x” years, and then to identify the number of years in full health that they consider to be of equal value [16]. For example, if a respondent considers that living for six years in full health is equivalent to being confined to a wheelchair for ten years, the utility that that person assigns to the state of being in a wheelchair can be used to calculate their QoL in that state of health. Alternatively, rating-scale methods, such as visual analogue scales, involve asking subjects to rate their state of health on a scale from 0 to 100, ranging from worst to best health imaginable. The second type of methods for assessing HRQL involves indirect procedures. These use health status instruments such as the Health Utilities Index [17] or the EuroQol [18]. None of these methods requires subjects to self-assign a QoL score; rather, an index is estimated on the basis of their responses to questions about different dimensions of their health, including mobility and pain [19, 20].

This study is conducted in Catalonia, a high-income Spanish region, and focuses on a population that is currently undergoing an accelerated ageing process. According to information released for 1990 by the region’s official website, 176,565 individuals were then aged 80 and above, that is, 2.9% of the whole Catalan population. More recently, in 2016, the size of this oldest-old age group had more than doubled, and today it represents 6.0% of the overall population [21]. Under an average scenario, demographic projections indicate that this rate is set to reach almost 10.0% by 2040. Aside from the challenges this represents for the public pension system [22], from a healthcare perspective this process highlights the need for optimal resource management, along with well-designed prevention and promotion actions.

Within this demographic context, several cross-sectional [23–25] and longitudinal [26, 27] studies examine the correlates of HRQL in older adults, but little is known about the oldest-old population. We aim to fill this research gap by focusing on a twofold objective with regards to the population aged 80 and over. First, we seek to develop a model that can provide a better understanding of the way in which demographic, educational, health and lifestyle variables correlate to HRQL. Second, we aim to identify those factors frequently linked to poor ageing that can actually be modified in earlier stages of life.

## Methods

### Data

We conducted a cross-sectional study in Catalonia (Spain), a region with 7.5 million inhabitants, whose capital and largest city is Barcelona. Our microdata are drawn from the Catalan Health Survey (ESCA) [28], which provides extensive information on individuals’ health-related behaviour and state of health, in relation to a set of sociodemographic variables. This official survey was first conducted in 1994 and since 2010 it has been repeated on a twice-yearly basis. The sample uses a random complex design, with strata based on age, gender and geographical area [29]. Personal interviews are used to collect the data, and the questionnaires used in each time-period are designed to be comparable.

To obtain a sufficiently large sample size, we used data made available over the last six years (2011 to 2016). Our final sample comprised a randomly selected population of 2264 home-dwellers (1025 males and 1239 females) aged 80 years and above. In 42.6% of the cases, the interviewer was unable to locate the designated sample individual. However, following the ESCA standard procedure, he/she was provided with a substitute that was identical to the original in terms of their stratification variables. In addition, 567 interviews were conducted through an indirect informant, owing to problems of ill-health or other impediments presented by the selected respondent. The overall final response rate was 100%.

### Measures

EuroQol-5D was used to assess the HRQL of our population of individuals aged 80 and over. This standardized instrument was developed in Europe in 1990 by the EuroQoL Group [18] and has since been widely used. It comprises two parts – an indirect and a direct procedure for assessing HRQL: that is, a descriptive health classifying system (EQ-5D) and a visual analogue scale (EQ-VAS), respectively. The EQ-5D is a self-report questionnaire that asks respondents to classify their own health according to five dimensions: mobility, self-care, usual activities, pain or discomfort, and anxiety or depression. Since 2011, the ESCA questionnaire has incorporated the five-level Spanish version [30], with five degrees of severity ranging from no problems to extreme impairment [31]. Additionally, the EQ-VAS, a rating-scale method, allows respondents to provide a global assessment of their health status “today”, from 0 to 100. Here, we opted to group the EQ-VAS values into four intervals delimited by quartiles, ranging from worst to best HRQL: i) interval 1: ≤ 40; ii) interval 2: 41 to 55; iii) interval 3: 56 to 70; and iv) interval 4: > 70. The EuroQol Group recommends the use of both parts of the instrument for research purposes [32].

We also took into consideration several sociodemographic, health status, and healthcare variables associated with HRQL. The sociodemographic characteristics included gender, age (three groups: 80–84; 85–89; 90-plus), level of education (low – less than high school vs. middle/high – upper secondary or tertiary), social class based on the classification proposed by the Spanish Society of Epidemiology [33] (low vs. middle/high social class), and household size (lives alone or lives with other household members). Levels of sensory and physical limitation were included by drawing on several different indicators. Specifically, the severity of sensory impairment was constructed by summing the number of affirmative responses when reporting the presence of limitations in: (i) hearing; (ii) seeing; (iii) speaking; and (iv) writing or reading. The level of functional loss was likewise constructed from affirmative responses indicating: (i) severe limitations in getting out of the house alone; (ii) walking problems that require mobility aids, the help of another person, or a wheelchair; and (iii) other physical movement impairments, such as difficulties in climbing ten steps without help. Chronic morbidity was measured through the number of self-reported chronic conditions, with subjects being asked if they currently have or had had various conditions selected from a list of 32, including hypertension, hypercholesterolemia, diabetes, etc. We also considered the occurrence of falls in the previous 12 months, the need for help to complete activities of daily living (ADLs), and the level of physical activity (from sedentary to highly active). A range of variables has typically been used as indirect controls of health indicators and to help classify individuals into a specific category. As risky health behaviours, we considered the following variables: body mass index (underweight or obese, that is, a BMI of < 18.5 or ≥ 30 vs. normal or overweight, that is, a BMI of ≥18.5 or < 30), smoking (current smoker, former smoker or never smoked), and drinking (non-drinker; moderate drinker, that is, drinking less than 280 g of pure alcohol per week for men, and 170 g for women; or at-risk drinker, when these limits are exceeded, or alcohol use is over 50 g in 24 h at least once a month). These alcohol intake categories were defined according to the classification provided by the Spanish Society of Family and Community Medicine [34]. As for healthcare and the use of medical resources, respondents reported about the number of prescription drugs taken during the previous two days, the number of overnight stays in a hospital within the preceding 12 months, and the number of visits or calls to the emergency department during the same period.

### Statistical analysis

We analysed the descriptive data of the sociodemographic characteristics and health status of Catalan adults aged 80-plus in relation to the EuroQoL instrument. Specifically, we provide the severity distribution for each dimension on the EQ-5D questionnaire, and the individual characteristics according to the quartile interval on the EQ-VAS. Continuous variables are described using means and standard deviations (SD), and categorical variables using percentages (%). Sampling weights provided by the ESCA are used in the analyses to correct for age and gender deviations between our sample and the Catalan population.

A multivariate linear regression model was fitted using ordinary least squares (OLS), to detect which components affected the EQ-VAS score for HRQL. To this end, we estimated and compared three different regression models. In a first step, Model 1 estimated the effect of the EQ-5D scale measures on the EQ-VAS score to determine their influence. Specifically, we wished to analyse the relationship between the score recorded by the study population on the EQ-VAS and their perception of the degree of severity of each of their limitations included in the EQ-5D. Our objective in so doing was to determine whether there are dimensions or degrees of severity that are more closely associated than others with a decrease in HRQL. In a second step, we omitted the EQ-5D measures, and included all the control variables, so as to identify their effect on the EQ-VAS score (Model 2). We aimed to facilitate identification of the net association between these variables and the assessment of HRQL measured using this scale, which lies at the core of our study. Finally, to determine how the variables included in Model 2 interact with the EQ-5D dimensions, we combined Models 1 and 2 (Model 3).

We tested for heteroscedasticity and multicollinearity in the three sequential models and obtained mainly negative results. Only Model 3 was slightly affected by some correlation between the EQ-5D variables and the rest of the regressors. However, as the latter is a combined model, we decided to maintain all the explanatory variables. The outcomes of the OLS models are presented as coefficients with their respective *p*-values (significance levels at 1, 5 and 10%). All statistical analyses were conducted with Stata version 13.1.

## Results

### Univariate analysis

Table 1 shows the distribution of individuals according to the reported level of severity on the EQ-5D dimensions. The dimensions most frequently identified by the respondents as severe/extreme problems were limitations related to usual activities (26.2%) and mobility (24.6%). Indeed, mobility issues, together with pain/discomfort, affected about two-thirds of the population to some degree, although only relatively small proportions identified them as extreme problems (4.8 and 3.2%, respectively). In contrast, roughly two-thirds of individuals reported no limitations as regards self-care and anxiety/depression (60.4 and 63.1%, respectively).

**Table 1:** Distribution (%) and 95% confidence intervals for the EQ-5D dimensions by levels of severity. Individuals aged 80-plus

EQ-5D dimensions	Total sample (*N* = 2264)									
	No problems	CI 95%	Mild problems	CI 95%	Moderate problems	CI 95%	Severe problems	CI 95%	Extreme problems	CI 95%
Mobility	33.9	(32.0; 35.8)	17.6	(16.0; 19.2)	23.9	(22.1; 25.7)	19.8	(18.2; 21.4)	4.8	(3.9; 5.7)
Self-care	60.4	(58.4; 62.4)	11.3	(10.0; 12.6)	10.7	(9.4; 12.0)	7.3	(6.2; 8.4)	10.4	(9.1; 11.7)
Usual activities	45.0	(43.0; 47.0)	14.5	(13.0; 16.0)	14.3	(12.9; 15.7)	11.3	(10.0; 12.6)	14.9	(13.4; 16.4)
Pain/discomfort	33.0	(31.1; 34.9)	16.8	(15.3; 18.3)	29.2	(27.3; 31.1)	17.7	(16.1; 19.3)	3.2	(2.5; 3.9)
Anxiety/depression	63.1	(61.1; 65.1)	15.6	(14.1; 17.1)	13.6	(12.2; 15.0)	6.0	(5.0; 7.0)	1.7	(1.2; 2.2)

Source: ESCA 2011–2016

*CI* confidence interval

The study sample, of which 64.4% were women, presented a mean age of 85.9 years (SD 4.3), with slightly more than half falling in the youngest age group (80–84) (Table 2). The vast majority of respondents had not completed secondary education (86.5%) and were of low social class (75.5%), while almost three quarters (72.2%) reported not living alone. As for their health, 32.4% of the respondents reported sensory problems of some degree; 44.5% presented a severity level of 2 or 3 in terms of functional loss; and, the average number of chronic diseases reported was 7.2 (4.0 SD). About a fifth of respondents reported suffering a fall in the previous 12 months, while more than half reported needing help with activities of daily living (ADLs) and being sedentary (with just 35.6% being slightly active or more). The average number of prescription drugs taken over the previous two days was 4.1 (SD 2.6). In the case of the three risk factors studied, 83.1% reported a normal/overweight BMI, 78.6% never smoked, and almost all reported being non-drinkers (52.7%) or moderate drinkers (46.6%). Finally, 17.4% of the elderly had been hospitalized in the previous year, with an average of 0.7 emergency visits (SD 1.6).

**Table 2:** Individual characteristics according to the quartiles of the EQ-VAS score. Individuals aged 80-plus

	N	Total	I1	I2	I3	I4
EQ VAS – Intervals^a^	2264		≤ 40	41–55	56–70	> 70
Age in years, mean (SD)	2264	85.9 (4.3)	86.0 (4.6)	85.4 (4.3)	84.6 (3.9)	84.5 (4.4)
Age groups	2264					
80–84		52.0	42.8	48.8	57.5	59.9
85–89		31.6	34.4	34.8	30.0	26.4
90+		16.4	22.8	16.4	12.5	13.7
Sex	2264					
Men		35.6	29.7	30.7	38.9	44.7
Women		64.4	70.3	69.3	61.1	55.3
Level of education	2260					
Low		86.5	92.4	88.6	84.4	79.4
Middle or high		13.5	7.3	11.4	15.6	20.6
Social class	2221					
Low		75.5	80.4	77.3	75.4	66.6
Middle or high		24.5	19.6	22.7	24.6	33.4
Household size	2264					
Living alone		27.8	20.5	31.4	32.2	26.9
More than one member		72.2	79.5	68.6	67.9	73.1
Level of sensory loss	2264					
0		67.6	48.3	65.2	77.1	82.9
1		19.7	24.6	23.8	16.2	13.2
2		8.6	17.6	6.4	5.6	3.5
3		2.7	6.5	2.8	0.6	0.4
4		1.4	3.0	1.8	0.5	0.0
Level of functional loss	2264					
0		38.4	10.6	30.1	52.1	65.8
1		17.1	11.1	17.5	22.4	17.0
2		14.0	15.1	19.5	12.9	7.5
3		30.5	63.2	32.9	12.6	9.7
Number of chronic diseases, mean (SD)	2264	7.2 (4.0)	9.5 (4.0)	7.7 (3.6)	6.3 (3.5)	4.9 (3.2)
Falls in the previous 12 months	2264					
0		79.0	71.1	77.3	83.2	85.2
> =1		21.0	28.9	22.7	16.8	14.8
Needs help for ADL	2264					
Yes		51.5	87.2	60.3	32.7	20.6
No		48.5	12.8	39.7	67.3	79.4
Usual physical activity	1552					
Highly active		0.2	0.0	0.0	0.3	0.6
Moderately active		0.7	0.3	0.7	0.8	1.1
Slightly active		34.7	14.1	24.5	42.0	52.9
Minimally active		8.1	5.2	8.9	9.4	7.8
Sedentary		56.3	80.3	65.9	47.5	37.7
Number of prescription drugs in the last 2 days, mean (SD)	2264	4.1 (2.6)	5.3 (2.8)	4.5 (2.3)	3.6 (2.3)	2.9 (2.2)
Risk Factors						
Body mass index	2264					
Normal or overweight		83.1	77.0	77.6	87.8	91.1
Underweight or obesity		16.9	33.0	32.4	12.2	8.9
Smoking	1697					
Current smoker		2.8	3.2	2.4	2.3	3.4
Former smoker		16.6	12.2	16.7	23.1	19.8
Never smoked		78.6	84.5	80.9	74.5	76.8
Drinking	1697					
Non-drinker		52.7	65.5	57.0	50.8	39.9
Moderate drinker		46.6	34.5	42.3	48.5	58.8
At-risk drinker		0.7	0.0	0.7	0.8	1.3
Hospitalization in the last 12 months	2262					
Yes		17.4	28.8	18.8	10.7	10.4
No		82.6	71.2	81.2	89.3	89.6
Number of emergency visits in the last 12 months, mean (SD)	2257	0.7 (1.6)	1.3 (2.5)	0.7 (1.3)	0.5 (0.9)	0.4 (0.8)

Source: ESCA 2011–2016

Numbers are percentages (%) unless otherwise stated. SD: standard deviation. EQ-VAS: EuroQol Visual Analogue Scale. I1, I2, I3 and I4 represent the intervals delimited by the EQ-VAS quartiles

^a^Individuals with EQ-VAS score coinciding with a quartile value are assigned to the lower interval

Table 2 also shows the characteristics of the 80-plus population according to their EQ-VAS scores (broken down into four intervals delimited by quartiles). The first interval (those with the lowest HRQL score) comprised the largest proportion of women (70.3% vs. 55.3% in the fourth interval, reporting the highest scores), with the highest average age (86.0 vs. 84.5), the largest proportions with a low education level (92.4% vs. 79.4%) and low social class (80.4% vs. 66.6%) and the highest proportion living in a shared household (79.5% vs. 73.1%). As expected, the least advantaged group according to the EQ-VAS score reported the worse state of health in relation to all sensory, functioning and health variables, as well as the greatest use of health resources. The differences between intervals were especially notable in relation to reported levels of some sensory loss (51.7% in the first interval vs. just 17.1% in the fourth), the severest level of functional loss (63.2% vs. 9.7%), the average number of chronic diseases (9.5 vs. 4.9) and the need for help with ADLs (87.2% in the first vs. 20.6% in the fourth). BMI and lifestyles were healthier in the group with the highest HRQL scores. Differences between the first and fourth intervals were notable in the cases of sedentarism (80.3% versus 37.7%), being underweight or obese (33.0% versus 8.9%), and the proportion of non-drinkers (65.5% versus 39.9%). Likewise, while 28.8% of the elderly in the first interval had been hospitalized in the previous 12 months, with an average of 1.3 emergency visits, the corresponding figures for those in the fourth interval were 10.4% and 0.4. In short, a persistent pattern is evident with conditions improving across the board as we move from the first (lowest EQ-VAS score) to the fourth (highest EQ-VAS score) interval.

### Multivariate analysis

In the multivariate analysis stage, we estimated the linear regression coefficients for the EQ-VAS scores in three sequential models. As expected, the five standardized dimensions of the EQ-5D scale explained a large proportion (40.0%) of the variation of the EQ-VAS scores (Model 1, Table 3). We found a strong, significant negative association between severe/extreme problems in all five dimensions and the EQ-VAS scores. The expected value for the dependent variable fell as the severity of the disorder increased. By order of magnitude, advanced stages of mobility issues, pain/discomfort, and anxiety/depression were associated with the largest falls in the EQ-VAS scores, while limitations as regards self-care and usual activities presented a weaker correlation.

**Table 3:** Estimated linear regression models for the EQ-VAS score. Individuals aged 80-plus

Variables	Model 1		Model 2		Model 3	
	Coefficients	*p*-value	Coefficients	*p*-value	Coefficients	*p*-value
EQ-5D Dimension						
Mobility problems						
No problems	–				–	
Slight problems	−5.37	***			−5.16	***
Moderate problems	−8.32	***			−8.52	***
Severe problems	−10.32	***			−10.3	***
Extreme Problems	−13.61	***			−14.04	***
Self-care problems						
No problems	–				–	
Slight problems	−2.51	*			−2.11	
Moderate problems	−5.10	***			−5.07	***
Severe problems	−6.81	***			−6.31	***
Extreme Problems	−6.89	***			−6.22	***
Usual activity problems						
No problems	–				–	
Slight problems	−2.13	*			−1.44	
Moderate problems	−4.30	***			−3.24	**
Severe problems	−5.30	***			−3.78	**
Extreme Problems	−7.82	***			−6.24	**
Pain/Discomfort						
No problems	–				–	
Slight problems	−3.35	***			−3.04	***
Moderate problems	−6.57	***			−5.64	***
Severe problems	−9.27	***			−8.36	***
Extreme Problems	−15.08	***			−13.71	***
Anxiety/Depression						
No problems	–				–	
Slight problems	−5.30	***			−4.47	***
Moderate problems	−6.22	***			−4.69	***
Severe problems	−6.52	***			−4.16	***
Extreme Problems	−12.42	***			−10.71	***
Constant	72.10	***	83.40	***	52.73	***
Age			−0.23	**	0.27	***
Female [vs. male]			−2.40	***	0.38	
Low education [vs. middle/high]			−3.58	***	−2.58	**
Low social class [vs. middle/high]			−2.18	**	−1.44	
Sensory impairment (0–4)			−5.18	***	−1.97	***
Chronic conditions (0–33)			−1.32	***	−0.49	***
Underweight or obese [vs. normal or overweight]			−2.89	***	0.47	
Number of prescription drugs in the last 2 days			−0.67	***	0.21	
Hospitalization in the last 12 months			−3.62	***	−2.08	**
Number of emergency visits in the last 12 months			−0.89	***	−0.25	
Sample size (N)	2264		2212		2212	
R-squared	0.40		0.25		0.42	

Source: ESCA 2011–2016

*** *p* < 0.01; ** *p* < 0.05; * *p* < 0.10. Linear regression models were used to estimate the EQ-VAS scores. EQ-VAS: EuroQol Visual Analogue Scale. The reference categories appear in brackets

Model 2 provides a different approach, as it excludes the EQ-5D measures, and instead includes as regressors certain socio-demographic, morbidity, functional status and lifestyle variables that also contribute to explaining the EQ-VAS score. Being older, female, low educated and belonging to the low social class reduced the expected EQ-VAS score. Individuals presenting some level of sensory impairment showed reductions of around 5 units on our HRQL measure for each additional issue reported. A similar outcome was found for those with chronic conditions (− 1.32 for each clinical condition). Being underweight or obese also had a significant negative association (− 2.89). Similarly, individuals taking prescription drugs in the previous two days, or who had been hospitalized or attended emergency units in the preceding twelve months presented lower expected HRQL values. The R-squared coefficient for this model was 0.25. To complete the analysis, we proposed an additional model (Model 3), which merges Models 1 and 2, allowing us to determine whether the variables in Model 2 interact with the EQ-5D dimensions. This last model presented the highest R-squared value (0.42), suggesting a good explanatory capacity. In the case of the EQ-5D dimensions, we found similar results to those presented above, except in relation to self-care and usual activity limitations whose coefficients for slight problems were no longer significant. Age presented a significant positive coefficient, suggesting that the EQ-5D scale might underestimate the dependent variable when combined with the rest of the regressors. On the other hand, gender, social class, underweight or obesity, the number of prescription drugs taken in the last two days and the number of emergency visits in the last 12 months were no longer significant. This result might suggest that these characteristics provide redundant information when the EQ-5D dimensions are included in the model.

## Discussion

This study has aimed to further our understanding of the ways in which sociodemographic, educational, health and lifestyle variables correlate with the HRQL among community-dwelling individuals aged 80-plus in Catalonia, a region where life expectancy is high and continues to increase. In so doing, we used a rating-scale method, the EQ-VAS, to measure the HRQL. Our findings confirm the multifaceted nature of HRQL in old age. As expected, the five dimensions of the EQ-5D were found to explain a significant proportion of the variability detected in the responses of the oldest-old about their HRQL. However, the consideration of other individual characteristics proved relevant for better outlining the profile of the population at risk of poor ageing due to bad HRQL. Specifically, our results shed new light on how different social and lifestyle factors correlate with the HRQL of the oldest-old. First, in the population aged 80-plus, they seem to confirm the positive association between being female and older and having a poor HRQL [35]. But, more interestingly, we detected a persistent pattern whereby other factors typically defined in younger stages of the life course are strongly linked to HRQL in later life. Specifically, middle/high levels of educational attainment and middle/high levels of social class were associated with a better HRQL for individuals aged 80-plus. These findings are in line with studies conducted in countries with different cultures [25, 36, 37], pointing to the widespread influence of education and income as social determinants of both physical and mental health at different stages of life and also among members of the oldest-age group. In addition to countless benefits across the lifespan, a higher education is an obvious facilitator of a better occupation-based socioeconomic status and, according to our results, of a better HRQL during old age.

While palliating the physical and mental deterioration caused by a long life remains essential, efforts to increase awareness among the population of the importance of preparing for successful ageing at younger stages in the lifecycle might improve the QoL at very old ages. For example, an active lifestyle could help prevent mobility issues or ADL difficulties in later life. In short, components of both health care and broader social issues may contribute to promote the best ageing trajectories [38].

The findings from our study are of considerable interest as they provide empirical evidence regarding a highly relevant aspect of health for contemporary societies. However, a number of limitations should be noted that might affect our results. First, as the ESCA does not target individuals in nursing homes, our results cannot be generalized to the entire oldest-old population. Indeed, our respondents may well be younger than those living in nursing homes, the latter generally being individuals at a higher risk of morbidity and mortality. According to the Catalan Statistics Institute [39], the institution-dwelling elderly represented about 0.6% of the Catalan population in 2011, with one in every five individuals aged 90-plus living in a nursing home. Second, the ESCA questionnaire was answered indirectly (owing to impairment of sample unit) in 25% of cases. Even though sensitive questions are avoided in this indirect questionnaire, some response bias might exist. Third, as our results cover a 6-year period, there could be some concealed trends. However, we were obliged to work with this long period in order to have a large enough sample to guarantee the robustness of the results. Finally, our findings would be clearer if longitudinal datasets had been used, but no panel data for Catalonia – or for Spain – are available.

While we recognize these limitations, the analysis presented here also has some strengths. We should stress that we have analysed a large sample of individuals aged 80-plus. Moreover, the official nature of ESCA ensures the quality of the field work and guarantees a full response rate, thus avoiding the underrepresentation of any age strata. As discussed, few studies to date have examined the determinants of HRQL among the oldest-old. Indeed, to the best of our knowledge, this is the first study using Spanish data to analyse the determinants of HRQL for individuals aged 80-plus. Bearing in mind that Spain leads the European life expectancy (83.0 years at birth –with Switzerland–, and 9.9 years at age 80 in 2015 [40]), furthering our knowledge of its oldest-old population is of undeniable interest.

## Conclusions

The multifaceted nature of QoL in old age highlights the need to implement a more multidisciplinary approach to understanding its complexity. A closer consideration of such domains as psychological and social support needs should provide a better insight as to why one elderly individual feels well despite the natural deterioration associated with ageing while another, in apparently similar conditions, feels unwell.

## Abbreviations

ADL: Activities of daily living
BMI: Body mass index
EQ-5D: EuroQol five dimensions
EQ-VAS: EuroQol visual analogue scale
ESCA: Catalan Health Survey (Enquesta de Salut de Catalunya)
HRQL: Health-related quality of life
QoL: Quality of life
